# Differential expression of CD148 on leukocyte subsets in inflammatory arthritis

**DOI:** 10.1186/ar4288

**Published:** 2013-09-09

**Authors:** Richa K Dave, Amy J Naylor, Stephen P Young, Rachel Bayley, Debbie L Hardie, Oliver Haworth, David A Rider, Andrew D Cook, Christopher D Buckley, Stuart Kellie

**Affiliations:** 1 School of Chemistry and Molecular Biosciences, The University of Queensland, Cooper Road, St. Lucia, QLD 4072, Australia; 2 Institute for Molecular Bioscience, The University of Queensland, 306 Carmody Rd, St Lucia, QLD 4072, Australia; 3 Centre for Translational Inflammation Research, School of Immunity & Infection, University of Birmingham, Vincent Drive, Birmingham B15 2TT, UK; 4 Department of Medicine, University of Melbourne, Royal Melbourne Hospital, Royal Parade, Parkville, VIC 3050 Australia; 5 Australian Infectious Disease Research Centre, The University of Queensland, Cooper Road, St Lucia, QLD 4072, Australia

## Abstract

**Introduction:**

Monocytic cells play a central role in the aetiology of rheumatoid arthritis, and manipulation of the activation of these cells is an approach currently under investigation to discover new therapies for this and associated diseases. CD148 is a transmembrane tyrosine phosphatase that is highly expressed in monocytes and macrophages and, since this family of molecules plays an important role in the regulation of cell activity, CD148 is a potential target for the manipulation of macrophage activation. For any molecule to be considered a therapeutic target, it is important for it to be increased in activity or expression during disease.

**Methods:**

We have investigated the expression of CD148 in two murine models of arthritis and in joints from rheumatoid arthritis (RA) patients using real-time PCR, immunohistochemistry, and studied the effects of proinflammatory stimuli on CD148 activity using biochemical assays.

**Results:**

We report that CD148 mRNA is upregulated in diseased joints of mice with collagen-induced arthritis. Furthermore, we report that in mice CD148 protein is highly expressed in infiltrating monocytes of diseased joints, with a small fraction of T cells also expressing CD148. In human arthritic joints both T cells and monocytes expressed high levels of CD148, however, we show differential expression of CD148 in T cells and monocytes from normal human peripheral blood compared to peripheral blood from RA and both normal and RA synovial fluid. Finally, we show that synovial fluid from rheumatoid arthritis patients suppresses CD148 phosphatase activity.

**Conclusions:**

CD148 is upregulated in macrophages and T cells in human RA samples, and its activity is enhanced by treatment with tumour necrosis factor alpha (TNFα), and reduced by synovial fluid or oxidising conditions. A greater understanding of the role of CD148 in chronic inflammation may lead to alternative therapeutic approaches to these diseases.

## Introduction

Mononuclear phagocytes ingest and destroy foreign microorganisms and act as antigen-presenting cells, thereby regulating both innate and acquired immunity by the release of proinflammatory products in response to pathogen-associated molecular patterns (PAMPs) [1-3]. Such responses commonly involve protein phosphorylation mediated by the selective activation of protein tyrosine kinases (PTKs), with further modulation by tyrosine phosphatases (PTPs). However, perturbation of the balance between PTK and PTP activity may result in a failure of inflammation to resolve leading to life-threatening chronic inflammatory diseases [4-8].

CD148 (PTPRJ; DEP-1; PTPη; Byp or PTPβ-like tyrosine phosphatase) is a receptor PTP containing a single cytoplasmic phosphatase domain and an extracellular domain containing eight to twelve fibronectin type III (FNIII) repeats [9]. Detectable in a wide range of cells [10,11], its reduced expression in some malignant tumours, coupled with the reversion of the transformed phenotype when CD148 function is restored, implies a role as a tumour suppressor [12-14]. Identified substrates/binding partners include Src family tyrosine kinases (SFK) [15,16], platelet-derived growth factor (PDGF) β-receptor [17], hepatocyte growth factor (HGF) receptor[18], vascular endothelial growth factor (VEGF) receptor-2 [19,20] and the p85 subunit of PI3-kinase [21]. In haemopoietic cells, it appears that CD148 has a positive role in enhancing SFK signalling by dephosphorylating a negative regulatory phosphotyrosine [15,16,22]. In contrast, dephosphorylation of PDGF-β receptor HGF receptor and VEGF receptor-2 appear to result in attenuation of proliferative responses to PDGF, HGF and VEGF respectively. By inhibiting phosphorylation of p85, CD148 can inhibit PI3 kinase activation, thereby potentially inhibiting any PI3 kinase-dependent proliferative signals [21]. In addition, p120 catenin [23], occludin and ZO-1 [24] have been identified as substrates or binding partners in epithelial cells. CD148-mediated dephosphorylation of these proteins leads to modulation of cell-cell adhesions [24,25].

CD148 expression is upregulated by cell density in some epithelial cells [26], and it plays a role in fibroblast [27] and monocyte [28] migration. However, although macrophages have the highest expression levels of CD148 [29,30], relatively little is known about its regulation and function in these cells. CD148 expression is upregulated by lipopolysaccharide (LPS) and downregulated by colony-stimulating factor 1 (CSF-1) [31]. In T cells, CD148 is excluded from the immunologic synapse and is able to reduce T cell receptor signalling [15,32,33]. CD148 dephosphorylates the negative regulatory tyrosine in SFKs in a manner similar to CD45 [15,16] and is essential for *Legionella pneumophila *phagocytosis and effector translocation [34]. More recently, a distinct function for CD148 in neutrophil chemotaxis has been reported [35]. Here we show that CD148 expression is upregulated in murine models of rheumatoid arthritis and in human rheumatoid arthritis, and show that this is primarily a result of expression by monocytic cells in mice and in both monocytic cells and T cells in humans within the joint lesions. An understanding of the biology of phosphatases such as CD148 in macrophage-specific signalling cascades may enable the identification of key endogenous regulators of inflammation and therapeutic targets for inflammatory diseases.

## Materials and methods

### Patients, peripheral blood and synovial tissue and synovial fluid preparation

Samples from peripheral venous blood (PB) and synovial fluid (SF) were collected into preservative-free heparin. Peripheral blood lymphocytes (PBLs) were isolated as previously described [36]. Synovial tissue was taken at the time of joint replacement and snap frozen in liquid nitrogen. All 39 patients with rheumatoid arthritis used in this study fulfilled 1987 ACR classification criteria for rheumatoid arthritis (RA) [37]. Peripheral blood lymphocytes (PBL) and synovial fluid lymphocytes (SFL) were isolated as previously described [36]. SF was immediately centrifuged to remove cells and debris before storage in aliquots at -70°C. All ethical approval and patient consent where appropriate for the use of tissue samples taken at the time of this study was obtained from the Birmingham Research Ethics Committee (REC 2002/088 and LREC 5735).

### Quantitative real-time PCR

RNA extraction from joint tissues was performed using the RNeasy Mini Kit (Qiagen, Valencia, CA, USA) as per the manufacturer's instructions. RNA purity was ensured by an A_260_/A_280 _of at least 1.8. Genomic DNA contamination was removed using DNase (Ambion, Austin, TX, USA) and cDNA was generated using Superscript III (Invitrogen, Carlsbad, CA, USA), using oligo-dT primers (Geneworks, Adelaide, Australia). Specific cDNA was quantitated in triplicate using SYBR Green (Applied Biosystems, Foster City, CA, USA) in 20 μL reactions in a 96-well plate. Data was analysed using the ABI Prism software. Gene expression was determined relative to *hprt *(hypoxanthine-guanine phosphoribosyl transferase) mRNA using the comparative threshold method. Unless otherwise stated, error bars indicate the standard deviation of duplicate cDNA quantitations each from three independent preparations of cells and RNA extracts using triplicate quantitations in the same thermal run. Where indicated, significance values between samples were analysed by a paired *t *test. Primers (f, forward; r, reverse) used were as follows:

*mouse csf1r f: *CCAGAGCCCCCACAGATAA, *r: *AGCTTGCTGTCTCCACGTTTG;

*human csf 1 f: *CCTTCAGGAGCAGGCCCAAG *, r: *CCTTGCTCGCAGCAGGTCAG;

*mouse CD148 f *CAGTACAGTGAATGGGAGCACTGAC, r: GTCCGTATTCTGCCACTCCAACT;

*human CD148 *f: AGTACACACGGCCCAGCAAT, r: GAGGCGTCATCAAAGTTCTGC;

*mouse hprt *f: GCAGTACAGCCCCAAAATGG, r: AACAAAGTCTGGCCTGTATCCAA;

*human hprt f: *TCAGGCAGTATAATCCAAAGATGGT, r: AGTCTGGCTTATATCCAACACTTCG.

### Antibodies

Monoclonal hamster anti-mouse CD148 antibody was generated as described [11] and was a gift from Prof. Arthur Weiss (Howard Hughes Medical Institute, University of California, San Francisco). Monoclonal anti-human CD148 was neat supernatant from the IgG2a clone 8B11H3 [28]. Anti-CD3 (IgG2b clone UCHT-1 17 μg/ml was a gift from Prof. Peter Beverley. Antibodies were purchased from commercial sources: F4/80 and Mac-2 (Serotec, Kidlington, UK); CD68 (Becton Dickinson, Franklin Lakes, NJ, USA); CD90 (Dianova, Hamburg, Germany); von Willebrand Factor (vWF) (Dako, Glostrup, Denmark). CD11c (IgG1 clone BU15 1 in 100 ascitis fluid) was a locally sourced Birmingham University hybridoma. Secondary antibodies were goat anti-mouse IgG1-FITC, IgG2a-TRITC and IgG2b-Cy5 (Southern Biotech, Birmingham, AL, USA); biotin-conjugated goat anti-rat IgG (Serotec); biotin conjugated goat anti-hamster IgG (Pierce, Rockford, IL, USA) and donkey anti-rabbit anti-methyl coumarin (Jackson ImmunoResearch, West Grove, PA, USA).

### Murine collagen-induced arthritis model

Eight- to twelve-week-old DBA mice were injected intradermally with chick collagen II in Freund's complete adjuvant and boosted 21 days later. This model has been extensively described [38-40]. Groups of nine mice were immunised with collagen and scored for arthritis onset using a scale of 0 (normal) to 3 (joint distortion and/or rigidity) for each limb two to three times per week for up to 60 days [39]. Typically about 35% of limbs were unaffected and 65% affected. Of the affected limbs, 50% display a score of 3, as described previously [38-42]. Rear joints were excised, fixed in 4% paraformaldehyde for 48 hours and decalcified in 10% EDTA prior to processing for paraffin embedding. Five micron serial sections were cut for immunohistochemistry. Three limbs of score 3 and controls were taken at day 35 post immunisation and RNA was isolated and processed for qPCR.

### KRN murine model for arthritis

Arthritis was induced in male, six-week-old C57Bl/6 mice (Harlan, Indianapolis, IN, USA) by intraperitoneal injection of 100 μl serum from K/BxN mice (a generous gift from Mohini Gray) as described [43,44]. Mice developed polyarthritis within 48 hours of the injection and were sacrificed by cervical dislocation at the peak of inflammation, three days after disease onset. Disease was scored as described above. Limbs were dissected and processed as described above.

### Immunohistochemistry

Immunohistochemistry was performed as described previously [45]. Expression of CD148 and Mac-2 were examined in serial sections. Briefly, sections were deparaffinized and rehydrated. For CD148, microwave antigen retrieval was performed in citrate buffer pH 6.0 for 2 min and allowed to cool overnight. For Mac-2 no antigen retrieval was necessary. Sections were washed in Tris-buffered saline (TBS) blocked in 3% H_2_O_2 _then serum block (10% fetal calf serum (FCS) plus 10% normal serum (species of secondary antibody) in TBS) followed by primary antibody for 60 min. Sections were subsequently incubated for 30 min with a biotinylated F(ab')2 fragment of goat anti-rat or rabbit anti-hamster immunoglobulin (DakoCytomation, Glostrup, Denmark), followed by horseradish peroxidase (HRP)-conjugated streptavidin (DakoCytomation), developed with DAB chromogen (DakoCytomation), and counterstained with Mayer's haematoxylin. The specificity of the staining was confirmed by using matched isotype control antibodies. Sections were dried and mounted with Cytoseal (Stephens Scientific, Riverdale, NJ, USA). Slides were using an Olympus BX51 microscope with DP70 camera (Olympus, Tokyo, Japan).

### Immunofluorescence

Five micron sections of snap-frozen tissue were air-dried and fixed for 10 min in acetone at 4°C. Sections were blocked for 1 hour at room temperature with 10% w/v bovine serum albumin (BSA) and then stained overnight at 4°C with primary antibodies. Sections were washed in phosphate-buffered saline (PBS) and then incubated with secondary antibodies for 1 hour at room temperature. Nuclei in murine tissue were stained with 20 μg/ml Hoechst 33258 (Bis-benzimid H33258 Fluorochrom, Riedel De Haen AG, Buchs, Switzerland). Sections were mounted in 2.4% 1,4-Diazabicyclo(2.2.2)octane (Sigma-Aldrich, St Louis, MO, USA) in glycerol (Fisons Scientific, Loughborough, UK) pH8.6. With reference to controls, images were captured using a Zeiss LSM 510 confocal microscope (Carl Zeiss, Oberkochen, Germany).

### Flow cytometry

Analysis of cell surface molecules was performed using three-colour immunofluorescence as previously described [36]. PBL and SFL were stained with the antibody cocktail of CD4 PE-Cy5 (1/50), CD8 FITC (1/20) and CD45RO PE (1/50). The cells were positively gated on the CD45 RO^+ ^and then on CD4 PE-Cy5^+ ^or CD8 FITC^+^. The samples were analysed on an EPICS XL Flow cytometer (Beckman-Coulter Inc., Brea, CA, USA). Data was analysed using WinMDI v2.8 (Scripps Institute, La Jolla, CA, USA). Where indicated, significance values between samples were analysed by a paired *t *test.

### CD148 phosphatase assay

CD4 peripheral blood human T cells were activated using anti-CD3 and gamma-irradiated Epstein-Barr virus (EBV) B cells. On day 2 cells were harvested, washed and then exposed to fresh medium containing SF from RA patients diluted to 1:5 or 1:10. To measure CD148 phosphatase enzyme activity a similar protocol to one we developed for the related CD45 was used [46] Cells were washed and lysed and CD148 captured onto protein A/CD148 Ab-coated 96-well plate and PTP activity assayed after the addition of fluorescein diphosphate substrate. The reaction was followed for an hour. Each assay was performed in triplicate and corrected for background (no capture antibody in the wells). Experiments were done with eight different SFs and in triplicate. For hydrogen peroxide treatment, two days after activation CD4 peripheral blood human T cells were exposed to hydrogen peroxide for 60 mins, lysed and CD148 phosphatase assay performed as described. For tumour necrosis factor alpha (TNFα) treatment, 0.1 ng/ml fresh TNFα was added daily for six days after which cells were lysed and CD148 phosphatase assay was performed. Where indicated, significance values between samples were analysed by paired *t *test.

## Results

### CD148 is upregulated in murine collagen-induced arthritis

**Figure 1:**
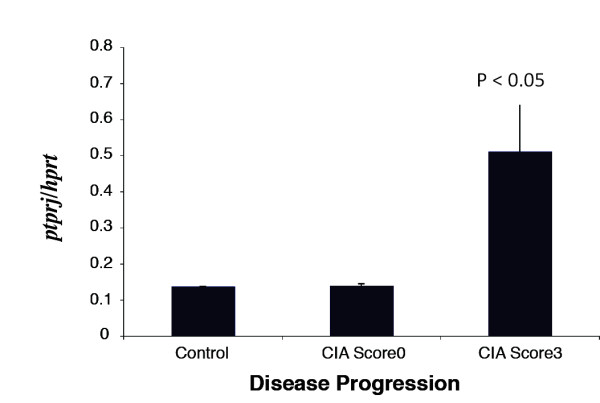
**CD148 mRNA in mouse collagen-induced arthritis model**. RNA was extracted from whole rear paws from the CIA mouse model, reverse transcribed and used to assess the expression of CD148 by quantitative real-time PCR. CIA score 0 refers to normal joints and 3 to severely inflamed joints marked by rigidity or joint distortion. The axis shows the relative expression of *ptprj *(CD148) compared to *hprt*, a control housekeeping gene. Data points (+/- SD) represent the average of triplicate samples each from triplicate independent experimental tissues, *n *= 3. CIA, collagen-induced arthritis; PCR, polymerase chain reaction.

Regulated expression of CD148 in macrophages in response to inflammatory stimuli *in vitro *strongly suggests a role in inflammation [31]. To investigate the relevance of these findings in an *in vivo *disease model, the expression of CD148 mRNA (*ptprj*) in collagen-induced arthritis (CIA) [39,45] was studied. Each mouse limb was scored for arthritis using a scale of 0 (normal) to 3 (joint distortion and/or rigidity and swelling) as described [39]. Real-time qPCR indicated that CD148 mRNA (*ptprj*) expression was elevated approximately five-fold in tissue from joints of mice that had severe arthritis (score 3) compared with mice with a score of 0 (Figure 1).

**Figure 2:**
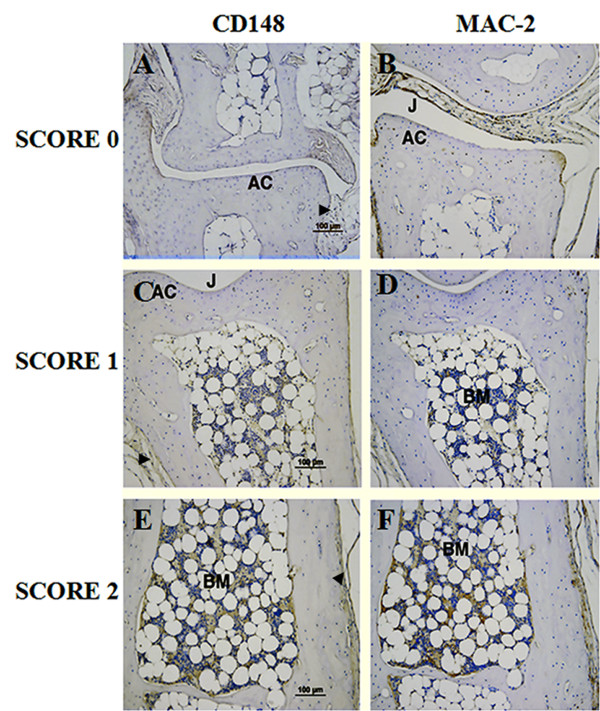
**Immunohistochemical detection of CD148 in joints from mice with differing collagen-induced arthritis severities**. Immunohistochemical staining for CD148 **(A, C, E) **and Mac-2 **(B, D, F) **protein expression (brown) was performed on joint tissue sections from CIA-immunized mice with score 0 (A, B), score 1 (C, D) and score 2 (E, F). All sections were counterstained with haematoxylin. AC, articular cartilage; BM, bone marrow; CIA, collagen-induced arthritis; J, joint space. Arrowhead denotes synovium. Magnification: x200; bar = 100 μm.

**Figure 3:**
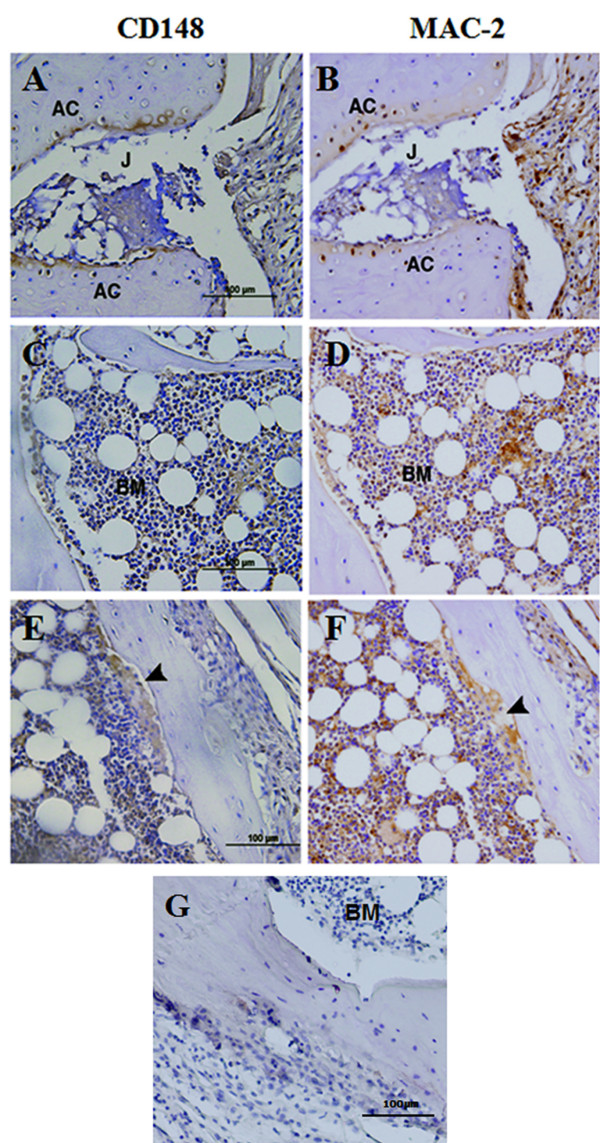
**Immunohistochemical staining of score 3 collagen-induced arthritis joints for CD148**. Anti-CD148 **(A, C, E) **and Mac2 staining (**B, D, F) **in score 3 joints. AC, articular cartilage; BM, bone marrow; G, isotype control nonimune IgG staining of score 3 joint; J, joint space. Arrowhead depicts area of bone resorption. Magnification: x200 (A-D); x400 (E-G); scale bar = 100 μm.

To determine whether there was any correlation between CD148 expression and disease pathology, immunohistochemical studies of CD148 in CIA joint tissue were performed. In normal joints (score 0), Mac-2 staining (a marker of inflammatory macrophages) was sparse in the synovium and was restricted to chondrocytes in the cartilage (Figure 2B). As expected, disease progression and severity was associated with an increase in immunoreactivity for Mac-2 (Figure 2D, F). In the normal joint few cells showed immunoreactivity to CD148 (Figure 2A). Score 1 inflamed joints showed little elevation in either CD148 or Mac2 staining (Figure 2C, D), however, score 2 joints displayed elevated levels of Mac2 and a concordant increase in CD148 staining (Figure 2E, F), indicating infiltration of inflammatory macrophages in the bone marrow of inflamed joints. CD148 expression was markedly elevated in score 3 severely inflamed joints (Figure 3A-F). Thus both CD148 and Mac-2 expression gradually increased with disease progression. There was a subpopulation of Mac-2 positive cells that were negative for CD148, therefore, not all infiltrating macrophages expressed CD148. Higher power imaging demonstrated that in score 3 joints CD148-positive cells were present in the synovial lining, inflamed joint space, bone marrow and in areas of tissue destruction and bone resorption (Figure 3). Compared to normal joints CD148 expression was highly upregulated in articular cartilage (Figure 3A) and in the bone marrow (Figure 3C). CD148 was strongly associated with areas of bone resorption (Figure 3E, F; arrowheads). Mac-2 staining on corresponding sections was indicative of the extent of inflammation. Therefore, these results were in accordance with the quantitative real-time PCR data showing increased CD148 mRNA expression (Figure 1) confirming that CD148 protein expression was elevated in CIA joint tissues, and identifying a subpopulation of macrophages that expressed CD148.

### Immunofluorescent localization of CD148 in two murine models of arthritis

**Figure 4:**
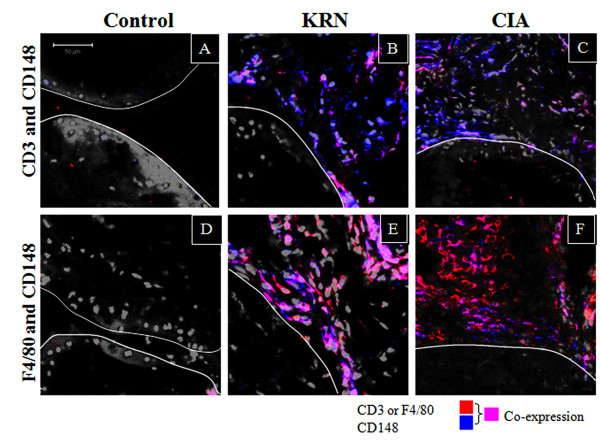
**Expression of CD148 is upregulated in two mouse models of arthritis**. Expression of CD148 is associated with T cells and macrophages. Two-colour immunofluorescence of CD148 with T cell marker CD3 **(A-C) **or macrophage marker F4/80 **(D-F) **in control unaffected animals, KRN arthritic and CIA arthritic mouse joints. CD3 or F4/80 (red), CD148 (blue), nuclei (grey). Co-expression appears as magenta. For each set of colours an example of a control, KRN and CIA joint is shown. The bone-synovial interface is marked with a white line for clarity. The images in this figure are representative of two experiments. All images are at the same magnification, scale bar is 50 μm. CIA, collagen-induced arthritis.

To further investigate the inflammatory cells expressing CD148, two-colour immunofluorescence was performed on joint tissue from both the CIA and the KRN models of experimental arthritis. Collagen-induced arthritis develops in some of the joints of mice after immunisation and boosting with collagen (CIA model). The KRN model utilises mice expressing the transgenic T cell receptor (TCR) KRN plus the MHC class II A^g7 ^(K/BxN mice). The advantage of this model is that the animals uniformly develop severe inflammatory arthritis, and serum from these mice reliably causes arthritis in many recipient strains. Development of disease requires the presence of T and B cells and autoantibodies to glucose-6-isomerase develop [43,44]. Tissue sections were stained for CD148 and either F4/80 (macrophage lineage) or CD3 (T cells). There was elevated staining of CD148 in both models of the disease (Figure 4). There was only limited co-staining of CD148 with CD3 in both the CIA and KRN mice (Figure 4B, C). However there was substantial co-staining of CD148 with F4/80 on both models (Figure 4E, F). Thus the majority of cells expressing CD148 in arthritic joints in mice in both inflammatory models were macrophages.

### CD148 is present in infiltrating cells in human arthritis joints

**Figure 5:**
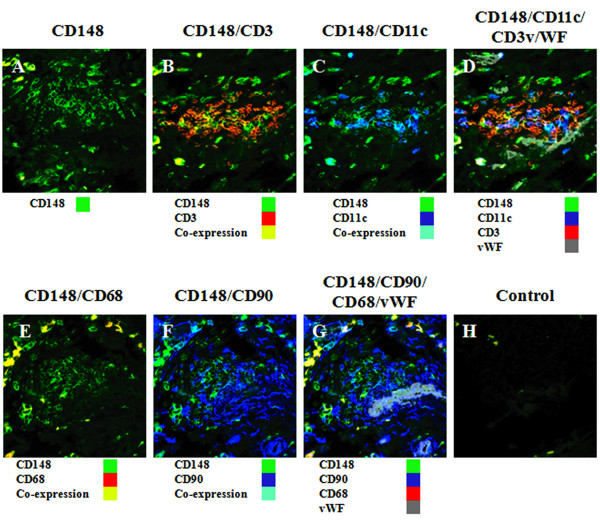
**Multi-colour immunofluorescence of CD148, CD68, CD11c, CD3, CD90 and von Willebrand factor in human rheumatoid arthritis joints**. Frozen sections of human RA joints were stained as described in Materials and methods. Four-colour immunofluorescence was performed with CD148 plus CD3/CD11c/vWF (panels **A-D**) or CD148 plus CD68/CD90/vWF (panels **E-G**). Panel **H **is a control with no primary antibody. RA, rheumatoid arthritis; vWF, von Willebrand factor.

To determine whether CD148 was present in synovial joints in human rheumatoid arthritis patients, immunofluorescence was performed with CD148 and CD68 (as a marker for macrophages); CD11c (as a marker for dendritic cells); CD3 (as a marker for T cells and/or thymocytes); CD90 (as a marker for stromal fibroblasts and endothelium); and von Willebrand factor (vWF) (as a marker for endothelial cells/blood vessels). CD148 was readily detectable in rheumatoid joints (Figure 5A). Four-colour immunofluorescence was used to determine which cells expressed CD148. A proportion of cells showed co-expression of CD11c and CD148, although the majority of cells were single positive for these proteins (Figure 5C). There was a subpopulation of cells that were double positive for CD148 and CD68 (Figure 5E), and a considerable number of cells were double positive for CD148 and CD3, although there were a number of CD148-positive/CD3-negative cells (Figure 5B). Endothelial cells, as identified by vWf, showed only low level staining for CD148 (Figure 5D, G). CD90-positive mesenchymal/stromal cells were generally negative for CD148 (Figure 5F, G). Taken together, we conclude that CD148 is preferentially expressed on T cells, macrophages and dendritic cells infiltrating the synovium (Figure 5, B, C, E).

### CD148 is differentially expressed in synovial and blood T cells and monocytes in rheumatoid arthritis patients

**Figure 6:**
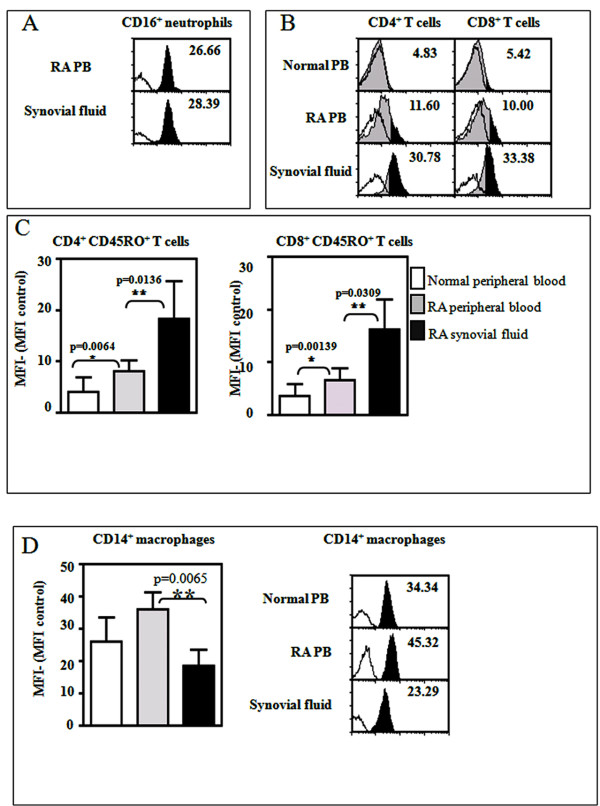
**Flow cytometry of CD148 in peripheral blood and synovial fluid cells of rheumatoid arthritis patients**. Cells were isolated either from peripheral blood or synovial fluid from RA patients or control patients as described in Materials and methods and subjected to flow cytometric analysis for CD148 and immune cell markers. **(A) **Flow cytometry of CD148 in PMNs from blood or synovial fluid of RA patient. **(B) **Flow cytometry of CD148 expression in CD8-positive or CD4-positive T cells (upper panels) from normal, RA patient blood or synovial fluid. **(C) **Quantitation of CD148 in CD8/CD45RO or CD4/CD45RO double positives. **(D) **Quantitation of CD148 expression in CD14-positive macrophages. In panels A, B and D clear areas under the curve are isotype control, grey shaded areas are anti-CD148, and filled black shading shows the expression of CD148 above isotype control. MFI, mean fluorescence intensity; PMN, polymorphonuclear neutrophils; RA, rheumatoid arthritis; *n *= 3; error bars depict SEM.

To quantify the expression of CD148 in different leukocyte subpopulations, flow cytometry was performed on peripheral blood cells from control and RA patients and from synovial fluid cells of RA patients. There was increased expression of CD148 in both CD4^+ ^and CD8^+ ^T cells in the peripheral blood of RA patients compared to controls (Figure 6B). When comparing T cells from the same patients, synovial fluid-derived CD4^+ ^and CD8^+ ^T cells had elevated levels of CD148 compared to the corresponding peripheral blood T cells (Figure 6B). Further analysis of CD4/CD45RO or CD8/RO double-positive cells showed a statistically significant increase in CD148 expression in synovial fluid-derived T cells compared with peripheral blood T cells, and a significant increase in CD148 expression in peripheral blood T cells from RA patients compared to peripheral blood T cells from control patients (Figure 6C). In contrast to this, there was a statistically significant decrease in CD148 expression in synovial fluid-derived CD14^+ ^macrophages compared with peripheral blood-derived CD14^+ ^macrophages from RA patients, whilst there was no significant difference in CD148 expression in peripheral blood macrophages from control or RA patients (Figure 6D). There was no significant difference in CD148 expression in peripheral blood-derived neutrophils or synovial fluid neutrophils from RA patients (Figure 6A).

### Synovial fluid from rheumatoid arthritis patients suppresses CD148 activity

**Figure 7:**
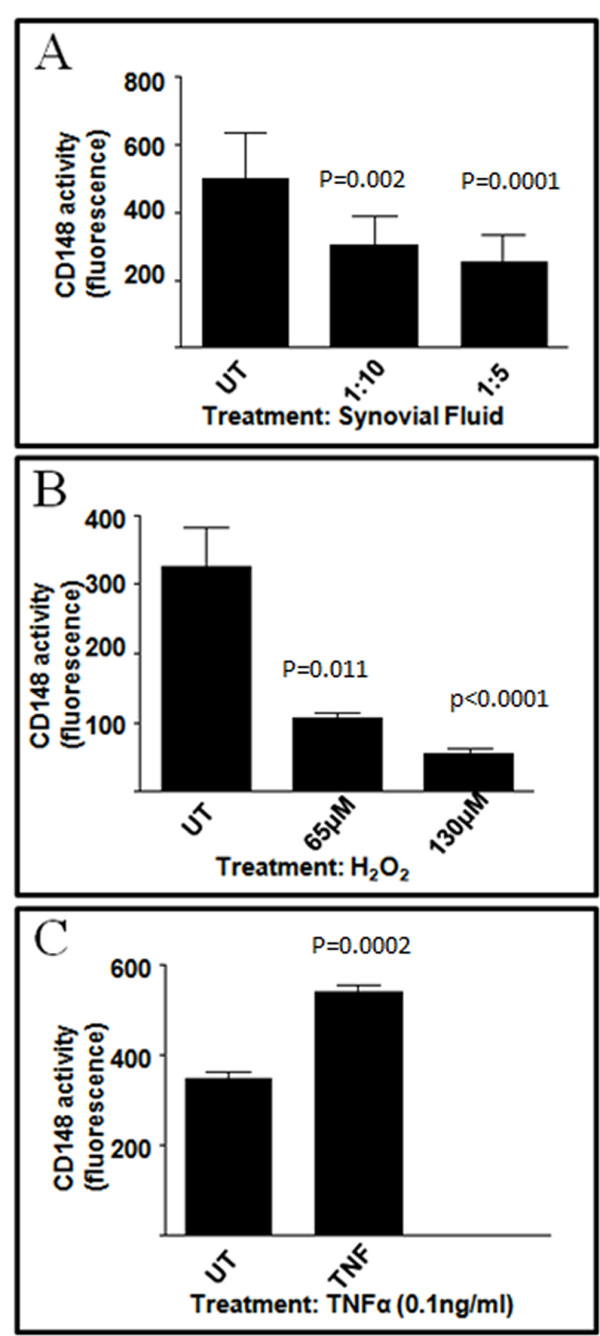
**Modulation of CD148 phosphatase activity in CD4^+ ^T cells by synovial fluid, hydrogen peroxide and tumour necrosis factor**. Human CD4^+ ^T cells were isolated and activated using anti-CD3 and gamma-irradiated EBV B cells. Two days after activation cells were treated with the indicated concentrations of synovial fluid **(A)**, hydrogen peroxide **(B) **or 0.1 ng/ml TNFα **(C) **as described in Materials and methods. CD148-associated tyrosine phosphatase activity was measured by a fluorescence assay as described in Materials and methods. EBV, Epstein-Barr virus; TNFα, tumour necrosis factor alpha. Bars indicate SEM; *n *= 8 (for panel A); *n *= 3 (for panels B and C).

Since CD148 was upregulated in CD4-positive T cells from rheumatoid joints, we asked whether the rheumatoid synovial environment might regulate CD148 function. CD4^+ ^peripheral blood T cells were isolated from normal volunteers and activated using anti-CD3 and gamma-irradiated EBV B cells. Cells were then treated with fresh medium containing SF from RA patients, or TNF. Alternatively cells were exposed to hydrogen peroxide for 60 min prior to lysis. CD148 was captured on 96-well plates coated with anti-CD148 and phosphatase activity measured [46]. SF significantly reduced the phosphatase activity of CD148 in a dose-dependent manner (Figure 7A). Short-term exposure to hydrogen peroxide also substantially reduced CD148 activity (Figure 7B). In contrast, 0.1 ng/ml TNF significantly elevated CD148 phosphatase activity (Figure 7C).

## Discussion

CD148 has been proposed to be a tumour suppressor in epithelial cells [47,48]. However, we show here that CD148 mRNA is highly expressed in macrophages and we further show that CD148 protein is upregulated in both experimental arthritis in mice and in human rheumatoid arthritis joints.

In murine arthritis qRT-PCR indicated increased levels of total CD148 mRNA in inflamed joints. Immunohistochemical studies revealed that the elevation in CD148 expression was strongly associated with Mac-2-positive inflammatory macrophages, suggesting a correlation between the extent of inflammation and CD148 expression. Activation of macrophages by TLR agonists induces expression of CD148 [29]. Since TLR engagement leads to nuclear factor kappa-light-chain-enhancer of activated B cells (NFkB) activation and is associated with an M1 phenotype, and CSF-1 (which downregulates CD148) is associated with an M2 phenotype, it appears that CD148 upregulation may correlate with an M1 phenotype. This is consistent with the presence of M1 mediators such as TNFα and IL1β in inflamed synovium. Immunofluorescence of human rheumatoid arthritis tissue also showed expression of CD148 in inflammatory joints, however in this case, CD148 expression was found mainly in infiltrating T cells with some expression in inflammatory macrophages. Quantification revealed that CD148 expression was increased in peripheral blood T cells from RA patients compared to control patients. Furthermore, in RA patients, synovial fluid-derived T cells had increased levels of CD148 compared to circulating T cells, again confirming that CD148 is differentially expressed in infiltrating inflammatory cells in RA joints. In contrast there were no differences in CD148 levels in circulating monocytes from RA patents compared with controls, and in fact there was a decrease in CD148 levels in synovial fluid-derived monocytes compared with circulating monocytes from RA patients. Our results are consistent with previous reports that mouse naïve T cells express little or no CD148, whereas human T cells express significant levels. There is no information on the expression of CD148 in different subpopulations of T cells or macrophages in arthritic diseases. The synovial environment plays a role in regulating cells activity [49,50]. Since immunofluorescence of human arthritic joints revealed that CD148 was primarily in T cells with lower apparent expression in macrophages, and quantification showed increased levels in T cells and lower levels of CD148 in macrophages from arthritic joints, we investigated whether the activity of CD148 in T cells might be modified by an inflammatory or SF environment. We found that SF from RA patients significantly reduced CD148 activity in CD4^+ ^T cells. SF is an oxidizing environment that also contains high concentrations of cytokines such as TNF, therefore we investigated the effects of these agents on CD148 activity. As expected, hydrogen peroxide (as an oxidising agent) inhibited CD148 activity, presumably by oxidizing the essential Cys1240 in the catalytic site of the molecule [51]. However, we also found that TNF increased CD148 activity in CD4^+ ^T cells. Thus, despite an increase in CD148 in isolated synovial T cells (Figure 6B, C), and high levels of TNF that, in isolation, would increase CD148 activity, we conclude that SF contains additional factors that counteract this to result in an overall inhibitory effect on CD148 activity. It is possible that SF contains other cytokines that suppress CD148 suppression, or other factors that maintain an oxidising environment. Since CD148 positively regulates T cell function we therefore expect that overall suppression of CD148 PTP activity in rheumatoid joints would suppress T cell activation. This may contribute to the observed lack of responsiveness of synovial T cells [49,50]. Whilst immunocytochemistry appears to show macrophage expression of CD148 in tissue joints (Figure 3), macrophages isolated from SF show a reduced expression of CD148 (Figure 6D). This is additional evidence that SF contains factors that influence CD148 expression in macrophages as well as in T cells as discussed previously.

CD148 expression is upregulated in other chronic inflammatory diseases such as Crohn's disease and Cogan's syndrome [52,53]. The possible function of CD148 in inflammation could be similar to that of CD45 [54-56] whereby it enhances T cell and BCR signalling by dephosphorylating the auto-inhibitory phosphotyrosine residues on SFKs [57,58]. Indeed, recent studies have revealed that CD45 and CD148 have overlapping functions and can activate SFKs in monocytes. CD148 negatively regulates neutrophil responses to chemokines by preferentially targeting the *lyn *tyrosine kinase and regulating G-protein-coupled receptor (GPCR) signalling [35]. The discrepancies between reports that CD148 can be a negative regulator or a positive of cell activation might be explained by the relative expression levels in cells. Recently, it has been shown that in endothelial cells, low-level expression of CD148 leads to promotion of responses by dephosphorylating Y529 of *c-src*, favouring VEGF-promoted proliferation. However, at higher expression levels, CD148 also dephosphorylates Y418 of *c-src*, attenuating downstream proliferation signals [20]. It is possible that there may also be differential thresholds for signalling pathway regulation in either T cells or macrophages. Two recent publications have proposed ligands for the extracellular domain of CD148. Thrombospondin-1 binds to the extracellular domain of CD148 with high affinity, and this activates its catalytic activity leading to dephosphorylation of substrates [59]. Syndecan 2 has been identified as another novel ligand for CD148, triggering activation of the C2β isoform of PI-3 kinase and subsequent β1 integrin-mediated adhesion [60]. It is interesting to note that both thrombospondin and syndecans are increased during inflammatory arthritis [61,62]. We have previously shown that an antibody against CD148 inhibits monocyte chemotaxis [28], thus there is growing evidence that CD148 can regulate cell motility. Both cell migration and adhesion are key markers of an inflammatory response, therefore differential activation of CD148 in inflammatory cells and a coordinate increase in endogenous ligand(s) could lead to an enhancement of the inflammatory response in migratory cells. The current results indicate that CD148 is highly regulated by inflammatory stimuli, however, the precise mechanism involved in the regulation of inflammation by CD148 remains to be elucidated.

## Conclusions

CD148 is a tyrosine phosphatase that has been implicated in cell growth and motility and its activity has been previously shown to enhance leukocyte activation by increasing SFK activity. We show here that in murine models of arthritis CD148 is upregulated in diseased tissue and is expressed at high levels in macrophages. In human RA CD148 is upregulated in both T cells and macrophages and in addition TNFα, a cytokine enriched in RA synovial fluid, can increase the tyrosine phosphatase activity of CD148. In contrast, both synovial fluid and an oxidizing environment reduce CD148-associated PTP activity. Thus CD148 in arthritic tissue can be regulated at both gene expression level and biochemical activity level. This regulation may results in enhanced cell activation in inflammatory lesions leading to an enhanced response. Whilst the current results indicate that CD148 is highly regulated by proinflammatory stimuli the precise mechanism underlying this regulation of inflammation remains to be elucidated.

## Abbreviations

CIA: collagen-induced arthritis; CSF-1: colony-stimulating factor 1; EBV: Epstein-Barr virus; HGF: hepatocyte growth factor; PAMP: pathogen-associated molecular pattern; PBL: peripheral blood leucocytes; PCR: polymerase chain reaction; PDGF: platelet-derived growth factor; PTK: protein tyrosine kinase; PTP: protein tyrosine phosphatase; RA: rheumatoid arthritis; SFK: Src family kinase; SFL: synovial fluid leukocytes; TNFα: tumour necrosis factor alpha; VEGF: vascular endothelial growth factor; vWF: von Willebrand factor.

## Competing interests

The authors declare that they have no competing interests.

## Authors' contributions

RKD performed the RT-PCR and immunohistochemistry of CIA joints. ADC performed the CIA model and scored and performed pathology on tissue sections. AJN, RB and DLH performed immunofluoresence and pathology on the KRN arthritis model. SPY, OH and DAR performed flow cytometry and biochemical tyrosine phosphatase assays on leucocytes from RA patients. SK and CDB devised the overall experimental strategy, supervised and organised the research. SK wrote the main draft. All authors contributed to writing the final draft and have read and approved the final document.
